# Intranasal immunization with outer membrane vesicle pertussis vaccine confers broad protection through mucosal IgA and Th17 responses

**DOI:** 10.1038/s41598-020-63998-2

**Published:** 2020-04-30

**Authors:** René H. M. Raeven, Dedeke Rockx-Brouwer, Gaurav Kanojia, Larissa van der Maas, Tim H. E. Bindels, Rimko ten Have, Elly van Riet, Bernard Metz, Gideon F. A. Kersten

**Affiliations:** 1 Intravacc (Institute for Translational Vaccinology), Bilthoven, The Netherlands; 2 Division of Drug Delivery Technology, Leiden Academic Center for Drug Research, Leiden University, Leiden, The Netherlands

**Keywords:** Vaccines, Mucosal immunology, Bacterial infection

## Abstract

A vaccine based on outer membrane vesicles of pertussis (omvPV) is protective in a mouse-challenge model and induces a broad antibody and mixed Th1/Th2/Th17 response against multiple antigens following subcutaneous immunization. However, this route did not result in mucosal immunity and did not prevent nasopharyngeal colonization. In this study, we explored the potential of intranasal immunization with omvPV. Only intranasal immunization induced strong mucosal immune responses that encompasses enhanced pulmonary and nasal IgA antibody levels, mainly directed against Vag8 and LPS. Furthermore, high numbers of IgA- and IgG-producing plasma cells were detected as well as lung-resident IgA memory B-cells. Finally, only intranasal immunization induced pulmonary Th1/Th17-related cytokine responses. The magnitude and type of systemic immunity was comparable between both routes and included high systemic IgG antibody levels, strong IgG-producing plasma cell responses, memory B-cells residing in the spleen and systemic Th1/Th2/Th17-related cytokine responses. Importantly, only intranasal immunization prevented colonization in both the lungs and the nasal cavity. In conclusion, intranasal omvPV immunization induces mucosal IgA and Th17-mediated responses without influencing the systemic immunity profile. These responses resulted in prevention of *Bordetella pertussis* colonization in the respiratory tract, including the nasal cavity, thereby potentially preventing transmission.

## Introduction

Immunization against the respiratory pathogen *Bordetella pertussis* resulted in a dramatic worldwide decrease of whooping cough cases^1^. However, the current pertussis resurgence occurs even in the vaccinated population, indicating that current pertussis vaccines or vaccination strategies should be improved^2,3^. Prolonged immunity is an important aspect for new pertussis vaccines as rapid waning of immunity is a major issue of current acellular pertussis vaccines (aPV)^4^. Moreover, it was demonstrated in baboons that aPV immunization prevents against disease but does not protect against transmission of *B. pertussis* to other baboons^5^. Nasopharyngeal carriage of *B. pertussis* in vaccinated individuals could be a potential cause for continuous spread by transmission^6^. Therefore, reducing nasal carriage by immunization is an important goal to prevent transmission and lowering the risk of exposure especially to unvaccinated individuals. Induction of mucosal immunity in the respiratory tract and particularly in the nasal cavity could assist preventing nasal colonization by *B. pertussis* and therefore reducing the chance of transmission^7^. *B. pertussis* infections induce powerful mucosal immunoglobulin A (IgA) and T helper (Th) type 17-mediated responses and prevent colonization in the complete respiratory tract upon reinfection^8,9^. In addition, the immune response after intranasal immunization with the live-attenuated pertussis vaccine BPZE1 is also characterized by Th17 and IgA responses and this vaccine diminishes the capability of *B. pertussis* to colonize the nose^10^. Mucosal immunity might therefore be an important mechanism to prevent nasal carriage and reduce the risk for transmission^7^.

Pertussis outer membrane vesicles (omvPV) are currently developed as a non-replicating vaccine candidate^11^ that provides protection against a *B. pertussis* infection in mice after intraperitoneal^12^ and subcutaneous immunization^13^. The protective immune response is characterized by a mixed Th1/2/17 response^13–15^ and a broad antibody response against multiple antigens such as *Bordetella* resistance to killing (BrkA), pertactin (Prn), autotransporter Vag8 and lipopolysaccharide (LPS)^16^, which are antigens that were all demonstrated to have protective capacity^17–20^. However, despite the excellent induction of systemic responses by systemic omvPV immunization, nasal carriage is not diminished. We recently showed that omvPV can be administrated directly in the respiratory tract leading to faster bacterial clearance from the lungs compared to subcutaneous immunization^15,21^. Pulmonary immunization also resulted in mucosal Th17 cells and IgA that were not present after subcutaneous immunization. In addition, pulmonary immunization evoked elevated systemic immunoglobulin G (IgG) antibody levels, IgG-producing plasma cells, memory B-cells, and Th17 cells as compared to subcutaneous immunization. While these data revealed the benefits of pulmonary over subcutaneous immunization with omvPV, the feasibility of pulmonary immunization is more challenging in terms of dose delivery, especially in the deeper lung area. Moreover, complete bacterial clearance from the nasal cavity was not achieved with pulmonary immunization. Intranasal immunization could serve as an alternative as the nasal cavity, the natural entrance site for pertussis, is an excellent site for vaccine delivery^22^ that would allow easier administration and could serve as a more efficient immunization site. Roberts *et al*. previously demonstrated that intranasal omvPV immunization leads to protection against a *B. pertussis* infection in the lungs^12^. However, the profiling of immune responses following intranasal immunization in a direct comparison with subcutaneous immunization are not yet reported in literature. In the current study, we investigated whether intranasal immunization with our omvPV concept provides protection against a *B. pertussis* infection, and in particular against nasal carriage. Additionally, systemic and mucosal antibody, B-cell and T-cell responses were studied to explore the underlying type of immunity.

## Materials and Methods

### Ethics statement

The animal experiment was carried out in accordance with the guidelines provided by the Dutch Act on Animal Experimentation. The animal experiment was approved by a local and independent ethical committee for animal experimentation of the Institute for Translational Vaccinology (Intravacc, Bilthoven, The Netherlands).

### Immunization and challenge of mice

**Figure 1:**
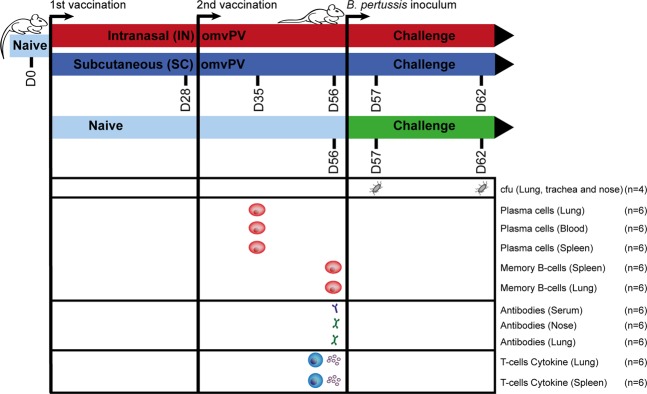
Study design. BALB/c mice (20 per group) were immunized with 4 µg outer membrane vesicle pertussis vaccine (omvPV) by the intranasal (IN; red) or subcutaneous (SC; blue) route on days 0 and 28. Subsequently, for both routes the vaccination-induced responses were characterized at day 35 and 56 (n = 6). Subsequently, a *B. pertussis* challenge (2 × 10^5^ colony-forming units (CFUs)) was performed on day 56 in both vaccinated groups and naive mice (green) after which the level of protection was assessed in the respiratory tract on day 57 and 62 (n = 4).

In a single experiment, 20 female, 8-week old BALB/c mice (Harlan, The Netherlands) were immunized twice (day 0 and 28) with 4 µg total protein omvPV for both administration routes, either administered via the intranasal (10 µL per nostril, total 20 µL), or subcutaneous route (300 µL) (Fig. 1). The *B. pertussis* challenge with the B1917 strain (2 × 10E5 colony forming units (CFU)) of immunized and naive mice (n = 4 per group, per time point) was performed on day 56 as described previously^15^.

### Sample collection

For immunological assays, mice were sacrificed at day 35 for analysis of plasma cells (n = 6 per group) and at day 56 for analysis of antibody, memory B-cell and T-helper responses (n = 6 per group). For colonization assays, mice were sacrificed at day 1 and day 6 post-challenge (n = 4 per group, per time point). Mice were anesthetized (isoflurane/oxygen) for orbital blood collection and sacrificed by cervical dislocation. (I) Serum for antibody detection was obtained by collecting whole blood in a serum collection tube (MiniCollect 0.8 mL Z Serum Sep GOLD, Greiner). After coagulation (10 min., room temperature), sera were collected by centrifugation (10 min., 3000 g), aliquoted and stored at −80 °C. (II) Whole blood for B-cell assays was collected in heparinized tubes (MiniCollect 1 mL LH, Greiner, Austria) after which erythrocytes were lysed using RBC lysis buffer (Pharm Lyse, BD). (III) Lungs and trachea for colonization assays were homogenized in 900 µL THIJS medium^23^ using a Bio-Gen PRO200 Homogenizer (Pro Scientific Inc., Oxford, USA). (IV) Nasal lavages for colonization assays were obtained by flushing the nose with 1 mL THIJS medium supplemented with 40 μg/mL cephalexin. (V) For B- and/or T-cell assays, complete lungs and spleens were collected in 5 mL RPMI complete medium (RPMI-1640 medium (Gibco) supplemented with 10% FCS (Hyclone), 100 units penicillin, 100 units streptomycin and 2.92 mg/mL L-glutamine (Invitrogen)) and homogenized using a 70-μm cell strainer (BD Falcon, BD Biosciences) by using a previously described protocol^15^. Spleen and lung tissue homogenates were treated with home-made lysis buffer (10 g/L NH_4_CL, 1.25 g/L NaHCO_3_, 0.125 mM EDTA in H_2_0; pH 7.4) to lyse erythrocytes. (VI) Lung homogenates and nasal lavages were sterile filtered with a 0.22 µm filter (Millex-GV, Millipore) and subsequently used for antibody detection at mucosal sites.

### Colonization assays

Lung and trachea homogenates and nose lavages collected at day 1 and day 6 post-challenge were serially diluted (undiluted, 1:10, 1:100, and 1:1000 depending on organ type) in THIJS medium. Subsequently, 100 µL of each sample was plated on Bordet-Gengou agar plates with 15% sheep blood (BD, The Netherlands) and incubated for 5 days at 35 °C. The number of CFU/mL was determined using a colony counter (ProtoCOL, Synbiosis, Cambridge, UK). The limit of detection for this method is 10 CFU/mL.

### Multiplex immunoassay (MIA) for antibody measurements

Antibodies against outer membrane vesicles (OMV), BrkA, fimbriae (Fim) 2/3, filamentous hemagglutinin (FHA), Prn, pertussis toxin (Ptx), and an autotransporter (Vag8) were measured using a MIA as described previously^15^. Serum samples were diluted 1:2000 for anti-OMV IgG and 1:100 for IgG (subclass) and IgA measurements. For measuring IgA levels, lung lysates were ten times diluted while nasal lavages were left undiluted. Data was acquired with a Bio-Plex 200, processed using Bio-Plex Manager software (v5.0, Bio-Rad Laboratories), and presented as fluorescence intensities (FI). The limit of detection of each analyte was set at 3x the background signal as detected in non-immunized mice.

### Western blotting for antibody profiling

The antibody profiling using SDS-PAGE and Western blotting to characterize serum IgG, pulmonary IgA and nasal IgA to identify immunogenic proteins was performed as described previously^21^. The antigen identification of immunogenic antigens was not done in this study but obtained from a previous study^16^. Two lanes containing a marker and a *B. pertussis* lysate were cut in pairs after the SDS-PAGE and blotting. These strips were incubated with individual samples. All blots were scanned at the same intensity. The markers were used to align the blots from different groups with each other. Subsequently, the marker of each blot was cropped off and only the blot on the lysate is depicted in the figures.

### B-cell ELISpot for plasma and memory B-cells

For analysis of memory B-cells, splenocytes and lung cells were stimulated (5 × 10E5 cells per well, 24-well plate; 5 days, 37 °C) with 10 μg/mL CpG ODN 1826 (Invivogen, San Diego, CA), 10 μg/mL pokeweed mitogen (Sigma-Alderich, Zwijndrecht, The Netherlands), and Staphylococcus aureus protein A of Cowan Strain (1:5000; Sigma) in RPMI complete medium with β-mercaptoethanol (1:25000; Sigma) to induce differentiation into antibody secreting cells^24^. The percentage of OMV specific antibody secreting cells were subsequently determined by ELISpot. The numbers of OMV-specific IgG- and IgA-producing plasma cells in blood, spleen and lungs were directly determined using the same ELISpot method, with 10 µg/mL wildtype B1917 OMV as coat, as described before^15^. Spots were counted with an AID iSpot reader (Autoimmun Diagnostika, Strassberg, Germany) and indicated as antibody-secreting cells per 5 × 10E5 cells.

### Cell stimulation and MIA for T-helper (Th) cytokine analysis

The single-cell suspension obtained from lungs and spleen was stimulated for 3 days with OMV (1.5 μg/mL) to induce cytokine production after which the supernatant was collected. The concentration of T-helper-related cytokines interleukin-4 (IL-4), IL-5, IL-10, IL-13, IL-17A, TNF-α and IFN-γ was determined in the supernatants using a ProcartaPlex Mix&Match Mouse 7-Plex (ThermoFisher). Data was acquired with a Bio-Plex 200 (Bio-Rad) and analyzed using Bio-Plex Manager software (v 5.0, Bio-Rad). Results were corrected for the background with an unstimulated control per mouse stimulation per cytokine and calculated in pg/mL. Statistical analysis was only performed on results where the average cytokine production per group was ≥2x altered in immunized groups compared to the naive group.

### Statistics

Data from antibody, cytokine, and colonization assays were log-transformed and statistically tested using a t-test. Significance of inter-group differences for B-cell ELISpot analysis was determined using a Mann-Whitney t-test. Significant p-values are indicated by *p < 0.05, **p < 0.01, ***p < 0.001, ****p < 0.0001.

## Results

### Bacterial clearance from respiratory tract

**Figure 2:**
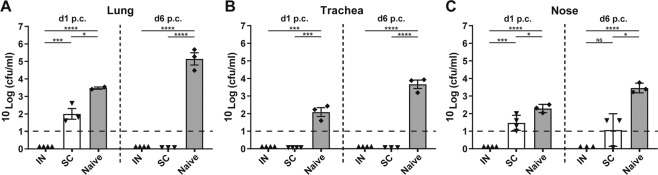
Colonization of the respiratory tract by *B. pertussis*. Naive and vaccinated mice were challenged with *B. pertussis* (2 × 10^5^ cfu) on day 56 and the number of cfu/ml were subsequently determined on day 1 (d57) and day 6 (d62) post-challenge (p.c.) in **(A)** lungs, **(B)** trachea, and **(C)** nasal lavages (mean with SEM and individual samples). The limit of detection (10 CFU/mL (Log1)) is depicted as horizontal dashed line. Significant differences are indicated by *p < 0.05, **p < 0.01, ***p < 0.001, ****p < 0.0001 obtained using a t-test after log-transformation of data. NS = not significant.

Protection against *B. pertussis* colonization of the respiratory tract was studied in mice that were immunized intranasally or subcutaneously with an omvPV. This was done by counting the number of viable bacteria, i.e. colony-forming units (cfu) in the lungs, trachea and nose on day 1 and day 6 post-challenge (p.c.) with *B. pertussis* (Fig. 2). The lungs, trachea and nose of naive mice were heavily colonized on day 1 after the *B. pertussis* challenge and the number of bacteria further increased on day 6 p.c., indicating successful colonization (Fig. 2A–C). In contrast, in the lungs and trachea of IN-mice, no cfu could be detected on both day 1 and day 6 p.c., whereas the lungs of SC-mice were not cleared 1-day p.c. (Fig. 2A,B). In the nose, only IN-mice were free of viable *B. pertussis* bacteria, whereas the mice in other groups showed presence of bacteria in the nasal lavages. However, the numbers of bacteria in the noses of SC-mice were lower compared to naive mice indicating some vaccine-induced protection (Fig. 2C). These results indicate that both subcutaneous and intranasal immunization with an omvPV enable faster bacterial clearance following a *B. pertussis* infection compared to non-immunized animals. However, full protection against colonization at all sites of the respiratory tract was observed with intranasal immunization.

### Mucosal humoral responses

**Figure 3:**
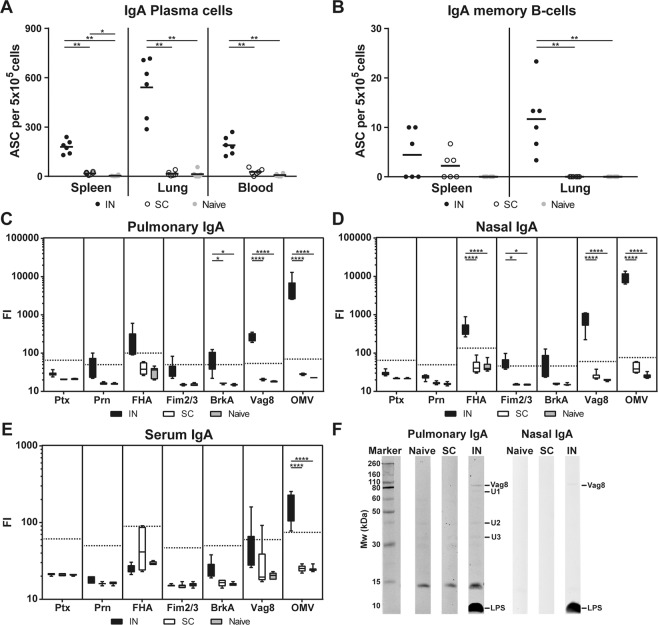
Mucosal humoral responses. (**A**) Numbers of OMV-specific IgA-secreting plasma cells in spleens, lungs and blood and **(B)** numbers of IgA memory cells, that were differentiated into antibody secreting cells (ASC), in spleens and lungs were determined by B-cell ELISpot of 6 mice per group at day 35 and day 56, respectively. Results are indicated as ASC per 5 × 10^5^ cells. Levels of immunoglobulin A (IgA) antibodies directed against Ptx, Prn, FHA, Fim2/3, BrkA, Vag8 and OMV were determined in (**C**) lungs, (**D**) nasal wash and (**E**) serum of 6 mice per group. Results are expressed as fluorescence intensities (FI). **(F)** Immunoproteomic profiles of pooled (n = 6) pulmonary and nasal IgA were determined using Western blotting using the same scan intensity. In the box plots, the box represent 25th to 75th percentiles, whereas the bars indicate the minimum and maximum value. Horizontal dashed lines represent the limit of detection (background x 3). Significant differences are indicated by *p < 0.05, **p < 0.01, ***p < 0.001, ****p < 0.0001 obtained using a Mann-Whitney t-test (B-cells) and t-test (antibodies) after log-transformation of data.

Mucosal humoral responses were investigated by analyzing (I) anti-OMV IgA-secreting plasma cells, (II) IgA memory B-cells and (III) IgA responses in the lungs, nasal wash and blood (Fig. 3A–F). IN-mice generated a significantly higher number of IgA-secreting plasma cells in the spleen, lung and blood compared to naive or SC-mice (Fig. 3A). Numbers of IgA memory B-cells in the lungs were significantly higher in IN-mice compared to SC-mice or naive mice, but there was no significant difference in the spleen (Fig. 3B). The levels of IgA antibodies directed against Ptx, Prn, FHA, Fim2/3, BrkA, Vag8 and OMV were determined in the lungs (Fig. 3C), nasal wash (Fig. 3D) and serum (Fig. 3E). Pulmonary IgA antibody levels directed against BrkA, Vag8 and OMV were significantly increased in IN-mice compared to SC-mice and naive mice (Fig. 3C). Nasal IgA antibody levels directed against FHA, Fim2/3, Vag8 and OMV were significantly increased in IN-mice compared to SC-mice and naive mice (Fig. 3D). In serum, only the anti-OMV IgA antibodies were significantly increased following intranasal immunization (Fig. 3E). Antibody profiling with Western blotting on a *B. pertussis* B1917 lysate demonstrated pulmonary and nasal IgA antibodies directed against LPS and Vag8 in the IN-mice but not in SC-mice or naive mice (Fig. 3F). The identity of these antigens was determined previously by mass spectrometry^16^. In addition, the pulmonary IgA of the IN-mice contained antibodies against three unidentified antigens (U1–3). These results indicate that the mucosal IgA humoral response was stronger after intranasal immunization as compared to subcutaneous immunization and that the IgA memory B-cells induced by intranasal immunization resided in the lungs.

### Systemic humoral responses

**Figure 4:**
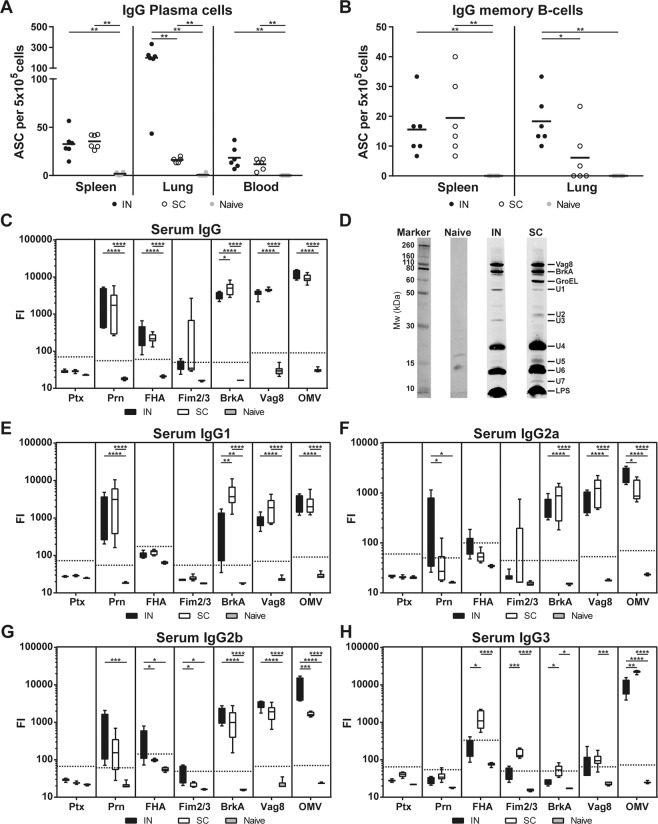
Systemic humoral responses. (**A**) Numbers of OMV-specific IgG-secreting plasma cells in spleens, lungs and blood and **(B)** numbers of IgG memory cells in spleens and lungs were determined by B-cell ELISpot of 6 mice per group at day 35 and day 56, respectively. Results are indicated as antibody-secreting cells (ASC) per 5 × 10^5^ cells. **(C–H)** Levels of **(C)** immunoglobulin G (IgG) antibodies and **(E–H)** IgG subclasses 1, 2a, 2b and 3 antibodies directed against Ptx, Prn, FHA, Fim2/3, BrkA, Vag8 and OMV were determined in serum of 6 mice per group. Results are expressed as fluorescence intensities (FI). **(D)** Western blot for antibody profiling with pooled (n = 6) serum IgG using *B. pertussis* B1917 lysate as antigen. The same scan intensity was applied to all blots. In the box plots, the box represent 25th to 75th percentiles, whereas the bars indicate the minimum and maximum value. Horizontal dashed lines represent the limit of detection (background x 3). Significant differences are indicated by *p < 0.05, **p < 0.01, ***p < 0.001, ****p < 0.0001 obtained using a Mann-Whitney t-test (B-cells) and t-test (antibodies) after log-transformation of data.

Induction of systemic immune responses was determined by measuring (I) anti-OMV IgG-secreting cells, (II) IgG memory B-cells and (III) IgG (subclass) antibody responses. OMV-specific IgG plasma cells were detected in spleen, lungs and blood of all omvPV-immunized mice, whereas these cells were not detected in naive mice, 7 days after booster immunization (Fig. 4A). Intranasal immunization resulted in a significantly higher number of anti-OMV IgG-secreting plasma cells in the lungs compared to the numbers observed in SC-mice. No significant differences in numbers of anti-OMV IgG-secreting plasma cells were observed in blood and spleen between the two immunized groups (Fig. 4A). Increased numbers of OMV-specific IgG memory B-cells were found in the spleens of both immunized groups (Fig. 4B). In addition, the IN-mice contained significantly higher numbers of IgG memory B-cells in the lungs compared to both SC-mice and naive mice (Fig. 4B).

In serum, the levels of IgG antibodies directed against BrkA, FHA, Prn, OMV, and Vag8 were significantly enhanced in both omvPV-immunized groups, when compared to naive mice (Fig. 4C). The levels of IgG antibodies directed against BrkA were slightly higher in SC-mice compared to IN-mice. A Western blot of anti-sera against bacterial lysate to establish the antigen specificity revealed overall a similar IgG response in intensity and diversity of immunogenic proteins between IN- and SC-mice. However, only the SC-mice induced additional antibodies against GroEL and the unidentified antigens U2 and U7, while the IN-mice had higher antibody formation against antigen U1 and exclusive antibody production against antigen U3 (Fig. 4D). The identity of the identified immunogenic proteins was determined previously by mass spectrometry^16^. Both SC- and IN-mice induced a broad IgG subclass response. Significant levels of IgG1, IgG2a, IgG2b and IgG3 antibodies against a variety of antigens were detected in vaccinated mice (Fig. 4E–H). Of note, the anti-Prn IgG2a levels were significantly lower in SC-mice when compared to IN-mice. The anti-BrkA IgG1 levels and anti-FHA IgG3 were slightly higher in SC-mice when compared to IN-mice. These results reveal that both subcutaneous and intranasal immunization induce similar, but not identical, systemic immune responses as measured in blood and spleen, while intranasal immunization resulted in elevated presence of IgG-producing plasma and memory B-cells locally in the lungs.

### Pulmonary and systemic T-helper cytokine responses

**Figure 5:**
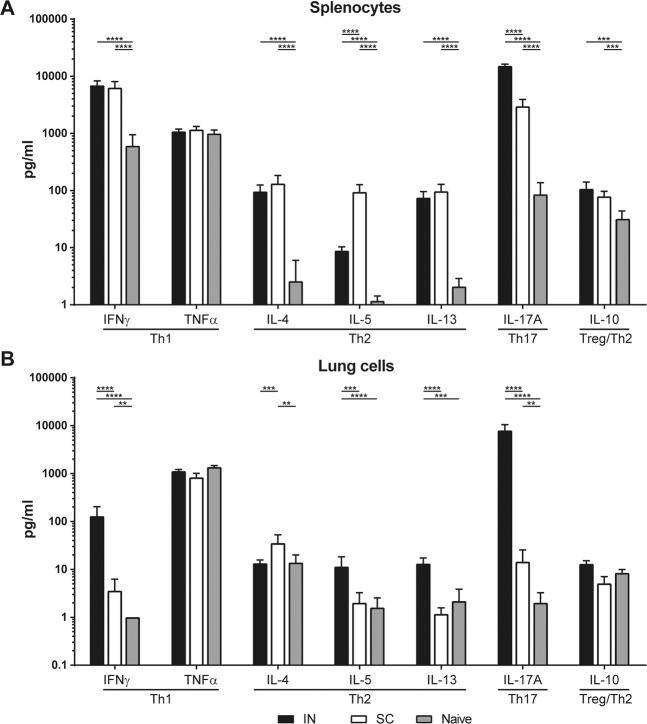
T-helper cytokine profiles in splenocytes and lung cells. Concentrations of T-helper 1, 2 and 17-related cytokines were determined in supernatant after 3-day stimulation with OMV of **(A)** splenocytes and **(B)** lung cells. Results in pg/mL are corrected for the background level (IMDM complete medium control) and are given as mean±s.d. of 6 mice per group. Significant differences (≥2x alteration compared to naive group) are indicated by *p < 0.05, **p < 0.01, ***p < 0.001, ****p < 0.0001 obtained using a t-test after log-transformation of data.

To determine the OMV-specific T-cell responses in spleens and lungs, cell suspensions from both organs were stimulated with OMVs for 3 days, after which concentrations of seven signature cytokines (IL-4, IL-5, IL-10, IL-13, IL-17A, TNF-α and IFN-γ) were determined in the supernatant by MIA (Fig. 5A,B). The Th17-related cytokine IL-17A was significantly increased in the supernatants of stimulated splenocytes and lung cells in both IN-mice and SC-mice compared to naive mice. This IL-17A production was however much higher in the IN-mice as compared to the SC-mice. The production of the Th1-related cytokine IFNγ was increased in lung cells of IN-mice compared to SC- or naive mice. In splenocytes, a similar increase in IFNγ production was observed in both IN- and SC-mice compared to naive mice. When looking at Th2-related cytokines, an increased IL-13 and IL-5 production was seen in the lungs of IN-mice that was absent in SC- and naive mice. In splenocytes, the Th2-related cytokines IL-4, IL-5 and IL-13 were significantly increased in SC- and IN-mice compared to naive mice. This IL-5 production was higher in SC-mice compared to IN-mice. Overall, these results demonstrate that both subcutaneous and intranasal immunization with omvPV result in a mixed Th1/Th2/Th17-related cytokine response, but that the intranasal immunization leads to additional induction of local Th1/Th17-related cytokine responses in the respiratory tract.

## Discussion

Current resurgence of pertussis, despite high vaccination coverage, occurs because protection induced by aPV is of limited duration^25^, is not effective against antigen-deficient (i.e. Prn-deficient) strains^26,27^ and fails to prevent *B. pertussis* transmission^5^. Therefore, the current focus for improving pertussis vaccines is on providing longer-lasting immunity, establishing adaptive immune responses against a broader range of antigens to increase the robustness of protection and prevention of transmission by reducing nasal carriage of *B. pertussis*.

Earlier we have shown that omvPV induces immune responses against a broad range of antigens, as well as a more Th1/Th17 and less Th2 biased response when administered subcutaneously or in the lungs^13,15,16,21^. In the current study, we compared intranasal and subcutaneous immunization with omvPV from the B1917 strain (summarized in Table 1**)**. Both subcutaneous and intranasal administration induced a Th1/Th17 polarized response and a broad humoral response in terms of multiple subclasses and against several antigens that were proven to be protective, such as BrkA^20,28^, LPS^17^ and Vag8^18^. These results indicate that the Th1/Th17 immune profile is related to omvPV, more than to the route of immunization. With respect to the antibody responses to these antigens, these were found to resemble antibody responses to natural *B. pertussis* infection in humans^29,30^ and mice^16^. These broad antibody responses induced by omvPV may provide protection through multiple antibody mechanisms such as opsonizing and bactericidal activity as well as prevention of bacterial adhesion^31,32^. However, the functionality of omvPV-induced IgG and IgA antibodies remains to be investigated.

**Table 1:** Comparison of immunity profiles induced by subcutaneous and intranasal OMV immunization.

		Naive	Immunization route for omvPV		Difference^***^
			Intranasal	Subcutaneous	
**Protection**	**Lung clearance**	>6 days p.c.	++++ (<1 day p.c.)	+++ (>1 day p.c.)	+
	**Tracheal clearance**	>6 days p.c.	++++ (<1 day p.c.)	++++ (<1 day p.c.)	0
	**Nasal clearance**	>6 days p.c.	++++ (<1 day p.c.)	++ (>6 days p.c.)	+++
**Systemic immunity**	**Antibodies**				
	Serum IgG	**−**	+ +++	+ +++	0
	Serum IgG1	**−**	++++	++++	+
	Serum IgG2a	**−**	++++	+++	+
	Serum IgG2b	**−**	++++	+++	+
	Serum IgG3	**−**	+++	++++	**−**
	Serum IgA	**−**	+++	**−**	+++
	Serum IgG specificity*	**−**	OMV, Vag8, BrkA, Prn, U1, U3, U4, U6, LPS	OMV, Vag8, BrkA, Prn, GroEL, U1–2, U4–7, LPS	0
	**Plasma cells**				
	IgG ASC Blood	**−**	+ +	+ +	0
	IgG ASC Spleen	**−**	++	++	0
	IgG ASC Lung	**−**	++++	++	++
	**Memory B-cells**				
	IgG ASC Spleen	**−**	+ +	+ +	0
	IgG ASC Lung	**−**	++	**−**	++
	**T-helper related cytokine responses**				
	Spleen Th1	**−**	+ ++	+ ++	0
	Spleen Th2	**−**	++	++	0
	Spleen Th17	**−**	++++	+++	+
**Mucosal immunity**	**Antibodies**				
	Pulmonary IgA	**−**	++++	+	+ ++
	Nasal IgA	**−**	++++	+	+++
	Pulmonary IgA specificity*	**−**	Vag8, LPS, U1–3 Vag8,	**−**	+++
	Nasal IgA specificity*	**−**	LPS	**−**	+++
	**Plasma cells**				
	IgA ASC Blood	**−**	+ +	**−**	+ +
	IgA ASC Spleen	**−**	++	**−**	++
	IgA ASC Lung	**−**	++++	**−**	++++
	**Memory B-cells**				
	IgA ASC Lung	**−**	+ ++	**−**	+ ++
	**T-helper related cytokine responses**				
	Pulmonary Th1	**−**	+ ++	+	+++
	Pulmonary Th2	**−**	+	**−**	+
	Pulmonary Th17	**−**	++++	+	++++

*Based on combined results of MIA and Western blotting. **Scale ranges from – (absent) to ++++ (highly present). ***Difference between intranasal and subcutaneous omvPV immunization. + to ++++ is advantage intranasal, 0 is no difference, **−** is advantage subcutaneous.

With respect to protection against colonization of *B. pertussis*, subcutaneous vaccination induced partial protection, whereas intranasal administration of omvPV provided full protection in the complete respiratory tract upon challenge. This is in agreement with the fact that mucosal immune responses such as IgA antibodies and tissue-resident memory B- and T-cells have generally been shown to play an important role in eliminating pathogens locally^33–36^. Also for pertussis vaccines, mucosal immunity may be beneficial to prevent transmission and limit residence time of the bacteria in the respiratory tract^6,7^. Intranasal immunization with experimental pertussis vaccines has been performed with multiple concepts such as a live-attenuated vaccine^10,37^, bacterium-like particles^38^, aPV with new adjuvants such as CpG^39^, LP-GMP^40^ or cholera toxin B^41^, omvPV from the Tohama strain^12^ and omvPV with detoxified LPS^42^. These studies demonstrate that intranasal immunization can prevent bacterial colonization in the respiratory tract in mice. Solans *et al*. demonstrated more specifically that the mechanism of their live-attenuated pertussis vaccine was depending partly on the induction of mucosal IgA responses and IL-17 producing tissue-resident memory T-cells^10^.

The profiling of underlying immune responses following omvPV immunization in the current study demonstrated that the most profound difference between intranasal and subcutaneous immunization was indeed the induction of strong mucosal responses (Table 1). Intranasal omvPV immunization provides strong mucosal IgA antibody responses against the potentially protective antigens Vag8 and LPS, and induces lung-resident memory B-cells (both IgA and IgG) and Th17-related cytokine responses. Local antigen encounter in the nasal cavity following intranasal immunization is required to evoke local responses such as memory B-cells^43^. Interestingly, despite administration at the outside of the mucosal barrier, intranasal omvPV immunization maintained a similar level of systemic IgG antibody levels, IgG-producing plasma cells, and spleen-resident memory B-cells as compared to subcutaneous omvPV immunization (Table 1). Both IgA-^44^ and Th1/Th17-mediated responses^45^ were previously identified as protective responses against *B. pertussis*. In line with intranasal omvPV immunization, similar mucosal IgA and Th17 responses were observed after a *B. pertussis* infection which provides excellent protection against a subsequent *B. pertussis* challenge^8,9,46^. Recent studies demonstrated that a *B. pertussis* infection also primes local innate immune cells, such as alveolar macrophages, M-cells and goblet cells^46^. These cells may also be induced by mucosal immunization with omvPV and could contribute to the faster bacterial clearance of the respiratory tract upon infection. Importantly, it was shown that an infection also induces tissue-resident T-cells in the lungs^47^. More research to whether these play a role in longevity of the memory response will be important for future research.

Previously, we demonstrated that induction of mucosal immunity and faster bacterial clearance from the lungs could be achieved through pulmonary administration of omvPV in both spray dried (reconstituted) and liquid form^15,21^. However, whereas the pulmonary immunization with omvPV led to significant reduction of *B. pertussis* in the nasal cavity, in the current study we demonstrated that intranasal immunization with omvPV provided complete *B. pertussis* clearance, also from the nasal cavity of mice. This difference cannot be explained by levels of nasal IgA, since these were similar and directed against the same antigens. In addition, in both cases pulmonary Th17 and IgA memory B-cells were detected^21^. However, possibly these administration routes result in qualitative differences in IgA, such as T-cell dependent versus independent induction, differences in the ratio of monomers, dimers and polymers or in levels and type of glycosylation of IgA antibodies, that can all affect IgA effector function. Alternatively, a head to head comparison could show whether the presence of local tissue-resident B- and T-cells in the nose could explain this difference.

In accordance with our previous findings^15,21^, the current study indicates that the intrinsic adjuvant activity present in the current form of the omvPV are suitable for inducing excellent systemic and mucosal immunity, obviating the need for additional adjuvants. In terms of safety, these intrinsic adjuvants may cause pyrogenicity. However, we observed earlier that systemic omvPV immunization induced less pro-inflammatory cytokines as compared to a whole-cell pertussis vaccine^13^. Due to the mucosal barrier, intranasal immunization may induce even less systemic side effects as compared to systemic immunization. However, to further lower endotoxic activity, an additional LPS modification can be inserted^48^. Insertion of the detoxified LPS, PagL, in an omvPV in combination with intranasal immunization did indeed further reduce pyrogenicity in mice^42^. Moreover, two studies reporting on safety in healthy individuals after intranasal immunization with vaccines against *Shigella sonnei*^49^ and *Shigella flexneri*^50^ based on outer membrane vesicles demonstrated that no serious adverse effects were observed. However, next to pyrogenicity, the local neurotoxic effects in the nose after intranasal omvPV immunization remain to be investigated clinically since this has been observed for *E. coli* heat-labile toxin adjuvanted influenza vaccine^51^. The cause of this neurotoxic effects were thought to be the combination of binding of de B subunit of LT to surface gangliosides (especially GM1) and inflammation associated with the A subunit. However, the use of the enzymatic A1 domain was shown to be safe and effective^52^.

With regard to human vaccination programs, the intranasal route offers a cost effective and patient friendly method for vaccine administration^53^. Our current findings could therefore justify further testing of different omvPV administration routes in other animal models such as baboons, as well as clinical trials and potentially in a human challenge model^54^. When used as a stand-alone booster, omvPV immunization could possibly reduce carriage of *B. pertussis* in the adult population, subsequently limiting the transmission to infants^55^. Additional research is needed to proof the hypothesis that intranasal administration might prevent carriage of *B. pertussis*. It would potentially even be more interesting when intranasal immunization with omvPV could be implemented as a primary vaccination to provide better protection against infection through induction of mucosal immunity and priming of children’s immune responses towards a more effective response^56^.

In conclusion, intranasal immunization of omvPV adds strong and broad mucosal IgA antibody responses, as well as lung-resident memory B-cells (both IgA and IgG) and Th17-related cytokine responses. This mucosal immunity is induced on top of the already promising systemic immune profile provided by subcutaneously administered omvPV that consists of a more Th1/Th17- and less Th2-related cytokine biased response and a broader antibody response (both subtypes and antigenicity) compared to aPV. Most importantly, intranasal administration prevented colonization of the lung, trachea and nose in the current study, which is important to prevent transmission, warranting further investigation in baboons or humans.

## Supplementary information

Supplementary Information.

## Data Availability

The authors declare that the data supporting the findings of this study are available within the paper.
