# Supplementary material for: Intranasal immunization with outer membrane vesicle pertussis vaccine confers broad protection through mucosal IgA and Th17 responses
Source: Sci Rep. 2020 Apr 30;10:7396. doi: 10.1038/s41598-020-63998-2 (PMC7192948; doi:10.1038/s41598-020-63998-2)
Supplement: Supplementary file 1 — Supplementary Information. [file 41598_2020_63998_MOESM1_ESM.docx]

**Supplementary information**

as part of

**Intranasal immunization with outer membrane vesicle pertussis vaccine confers broad protection through mucosal IgA and Th17 responses.**

René H. M. Raeven^a,^*****, Dedeke Rockx-Brouwer^a^, Gaurav Kanojia^a^, Larissa van der Maas^a^, Tim H. E. Bindels^a^, Rimko ten Have^a^, Elly van Riet^a^, Bernard Metz^a^, Gideon F. A. Kersten^a^**^,^**^b^

**^a^** Intravacc (Institute for Translational Vaccinology), Bilthoven, The Netherlands

^b^ Division of Drug Delivery Technology, Leiden Academic Center for Drug Research, Leiden University, Leiden, The Netherlands

*Corresponding author: Antonie van Leeuwenhoeklaan 9, P.O. BOX 450, 3720 AL Bilthoven, The Netherlands.

Tel. +31 30 7920 500. Email address: [rene.raeven@intravacc.nl](mailto:rene.raeven@intravacc.nl)





**Supplementary information 1** – Full length images of blots of sera for IgG responses and lung lysates and nasal washes for IgA responses.
